# Optimizing the implementation of lung cancer screening in Scotland: Focus group participant perspectives in the LUNGSCOT study

**DOI:** 10.1111/hex.13632

**Published:** 2022-10-20

**Authors:** Debbie Cavers, Mia Nelson, Jasmin Rostron, Kathryn A. Robb, Lynsey R. Brown, Christine Campbell, Ahsan R. Akram, Graeme Dickie, Melanie Mackean, Edwin J. R. van Beek, Frank Sullivan, Robert J. Steele, Aileen R. Neilson, David Weller

**Affiliations:** ^1^ Edinburgh Clinical Trials Unit, Usher Institute University of Edinburgh Edinburgh UK; ^2^ School of Health and Wellbeing University of Glasgow Glasgow UK; ^3^ School of Medicine University of St Andrews St Andrews UK; ^4^ Centre for Inflammation Research and Edinburgh Cancer Research Centre University of Edinburgh Edinburgh UK; ^5^ Care of the Usher Institute University of Edinburgh, Edinburgh Edinburgh UK; ^6^ Edinburgh Cancer Centre Western General Hospital Edinburgh UK; ^7^ Edinburgh Imaging, Queen's Medical Research Institute University of Edinburgh Edinburgh UK; ^8^ School of Medicine, Ninewells Hospital University of Dundee Dundee UK; ^9^ Present address: The National Institute of Economic and Social Research 2 Dean Trench Street, London NW1P 3HE UK

**Keywords:** early detection, focus group, lung cancer, lung screening, qualitative, screening

## Abstract

**Introduction:**

Targeted lung cancer screening is effective in reducing lung cancer and all‐cause mortality according to major trials in the United Kingdom and Europe. However, the best ways of implementing screening in local communities requires an understanding of the population the programme will serve. We undertook a study to explore the views of those potentially eligible for, and to identify potential barriers and facilitators to taking part in, lung screening, to inform the development of a feasibility study.

**Methods:**

Men and women aged 45–70, living in urban and rural Scotland, and either self‐reported people who smoke or who recently quit, were invited to take part in the study via research agency Taylor McKenzie. Eleven men and 14 women took part in three virtual focus groups exploring their views on lung screening. Focus group transcripts were transcribed and analysed using thematic analysis, assisted by QSR NVivo.

**Findings:**

Three overarching themes were identified: (1) *Knowledge, awareness and acceptability of lung screening*, (2) *Barriers and facilitators to screening* and (3) *Promoting screening and implementation ideas*. Participants were largely supportive of lung screening in principle and described the importance of the early detection of cancer. Emotional and psychological concerns as well as system‐level and practical issues were discussed as posing barriers and facilitators to lung screening.

**Conclusions:**

Understanding the views of people potentially eligible for a lung health check can usefully inform the development of a further study to test the feasibility and acceptability of lung screening in Scotland.

**Patient or Public Contribution:**

The LUNGSCOT study has convened a patient advisory group to advise on all aspects of study development and implementation. Patient representatives commented on the focus group study design, study materials and ethics application, and two representatives read the focus group transcripts.

## 1 INTRODUCTION

Lung cancer remains one of the major causes of cancer mortality globally, and Scotland has high incidence and low survival rates compared with the rest of Europe.^1^, ^2^, ^3^, ^4^ Despite many initiatives to raise awareness and encourage early symptomatic presentation, most lung cancers are diagnosed at a late stage, and overall survival is poor.^5^ A recent Public Health Scotland report revealed that the Covid‐19 pandemic has had a further negative impact on the clinical presentation of lung cancer, with a reduction in diagnoses during the lockdown periods, and an anticipated wave of late diagnoses from this backlog.^6^

Over the last 5 years, there has been a growing body of evidence from trials in the United States and continental Europe, and pilot studies in the United Kingdom, demonstrating survival gains from low‐dose computed tomography (LDCT) screening for lung cancer in high‐risk populations.^7^, ^8^, ^9^, ^10^ A recent meta‐analysis of this evidence provides a strong case for the implementation of targeted lung screening, supported by expert clinical opinion,^11^ and the UK National Screening Committee has recently recommended in favour of targeted lung screening. However, there are still many lung screening implementation challenges to be addressed.^12^, ^13^ Importantly, lung screening is most effective if targeted at high‐risk groups—ideally using validated risk prediction tools to assess who is at the highest risk of developing lung cancer and would most benefit from screening, namely smokers or recent quitters, aged 55–74 years.

To date, UK pilots and trials, including the Early Detection of Cancer of the Lung Scotland (ECLS) trial in Scotland using blood biomarkers to detect early signs of lung cancer, have shown variable and socially patterned uptake of screening,^8^, ^14^, ^15^, ^16^ and work has been done to understand barriers to participation. Uptake remains lower in more marginalized groups—that is, heavy smokers living in more deprived areas.^17^ Further research to understand the views of Scotland's population on the acceptability of lung screening, and barriers and facilitators (related to issues of geography, rural and urban deprivation and a high burden of multimorbidity), will help shape future pilots and programmes in Scotland.

This focus group study explores the views of people potentially eligible for lung screening, identifies perceived barriers and facilitators to participation, and examines strategies to optimize the implementation of lung screening in Scotland. It forms part of the LUNGSCOT study, which is examining the feasibility of introducing lung screening in Scotland.^18^

## 2 METHODS

We conducted three focus groups with self‐reported ‘heavy smokers’ living in rural, urban and deprived areas of Scotland to ascertain their views on barriers to lung screening. Focus groups were considered the most appropriate method for engaging with those eligible for screening, as they enabled an understanding of how participants perceive the prospect of lung screening through discussion and they enable the sharing and development of ideas.^19^ Findings from the focus groups will feed into the development and design of the lung screening pilot. We employed a range of strategies and reflexive practices to ensure that focus groups were participant‐led and data‐driven.^20^

### 2.1 Identifying participants

Participants were recruited through Taylor McKenzie (TM), a Scottish‐based company that specializes in qualitative research, to identify members of the public eligible to take part in the focus groups (https://www.taylormckenzie.co.uk). TM developed a study‐specific screening questionnaire (further details below) to allow the purposive sampling of eligible participants from their extensive database of people willing to take part in health, social or marketing research.

### 2.2 Recruitment and sampling

We aimed to recruit up to 24 people from across Scotland to take part in three separate focus groups of 8 people, considered to be an appropriate number to identify a range of views.^21^ Interested people responded to recruitment notices posted on TM's mailing list and social media pages. We used the inclusion and exclusion criteria listed In Box 1.

Box 1: **Inclusion and exclusion criterion**

Inclusion criteria

- Men and women
- Age 45–70 (inclusive)
- Current residence in Scotland
- Self‐identify as smoker or recent quitter (within the last 2 years)
- Able to undertake focus group interview in English
- Willing to discuss their views on lung screening

Exclusion criteria

- Lacking capacity to give informed consent
- Never smoker
- Smoker who quit more than 2 years ago
- Non‐English speakers, preventing them from comfortably taking part in a discussion

Those who responded were provided with a study information sheet by TM and given 7–14 days to consider taking part. TM drew up a list of eligible participants (based on information on their database, e.g., socioeconomic grade [SEG], occupation, and from speaking to potential participants directly, e.g., smoking status) and added it to a secure portal for the researchers to access and review. Eligible participants were allocated to a focus group at a preset date and time. Participants were offered a financial reimbursement for their time, paid via TM.

### 2.3 Focus groups and consent

Three focus groups, lasting approximately 75 min, were run virtually using the online video conferencing platform Zoom, selected due to likely participant familiarity with it, and to comply with the prevailing government restrictions on face‐to‐face meetings at the time due to the Covid‐19 pandemic. Participants were asked to sign a digital consent form via TM ahead of the focus group and verbal consent was agreed upon at the beginning of each focus group. Focus groups were led and facilitated by two researchers (D. C. and M. N.—health services researchers), with one group comprising those living in rural areas and the other two urban groups. Participants did not know one another before the focus group. The format and content of the focus groups were developed by a subgroup of the research team with expertise in behavioural aspects of cancer screening, drawing on relevant literature. The lung screening process was explained to participants: eligible people would be offered a LDCT scan to detect any lung conditions, one of which is lung cancer. The topic guide (see Supporting Information: 1) covered views on lung screening, with a particular focus on perceived barriers to taking part, personal resources to facilitate screening, understanding of the process, and input on what a good screening programme would look like. With consent, focus groups were recorded.

### 2.4 Analysis

Focus group recordings were transcribed verbatim and subject to Braun and Clarke's^22^ thematic analysis, chosen as it is the most commonly used approach considered appropriate to derive key themes and ideas from the group discussions, taking context into account^19^ and consistent with social constructionist underpinnings. Using thematic analysis allowed us to incorporate guidance specific to focus group interviewing and its impact on analysis, for example, considering dynamics, social comparison and power imbalances within groups.^23^, ^24^ Transcripts were read repeatedly and compared and contrasted to develop a set of common codes by D. C. These codes were applied back across the data and assigned to excerpts from the focus groups using QSR NVivo version 12 Pro (www.qsrinternational.com) by D. C. and J. R. (a research intern). Codes were further refined and a set of overarching themes and subthemes were inductively derived to interpret and explain the data, in discussion with the wider research team and patient advisory group. The LUNGSCOT team comprises health services researchers, health psychologists, clinicians, a health economist and patient and carer representatives. Themes were placed in the context of existing literature and theory to incorporate our findings into the wider evidence base.

### 2.5 Patient advisory group

The patient advisory group was convened for the purpose of the wider LUNGSCOT study. The group comprises three patients and one carer with experience in lung cancer and two patients with other cancers. The group has been involved in the study design and commented on study documentation as well as two advisors reading transcripts and sharing their views on the analysis. The group meets quarterly to discuss study progress and opportunities to get involved in study tasks.

## 3 FINDINGS

Eleven females and 14 males aged 45–70 years living in a mix of urban and rural areas in Scotland took part across the three focus groups. Eleven participants were current smokers, and 14 had quit within the previous 2 years. All participants were from the lower socio‐economic grades (SEG): C2 (skilled manual workers), D (semiskilled and unskilled manual workers) and E (nonworking). Twenty‐one participants were White British/Scottish, one person was Black British, one British Asian and one South Asian. All participants had school‐level or vocational qualifications but no one had a higher education degree. See Tables 1, 2, 3 for participant characteristics.

**Table 1:** Focus Group 1 participant characteristics

Focus group 1	Gender	Age range	NHS Health Board	Urban/rural area	SEG	Smoking status
Participant (P)1	M	45–49	Greater Glasgow and Clyde	Urban	C2	Current smoker
P2	M	60–64	Fife	Urban	D	Current smoker
P3	M	70–74	Forth Valley	Urban	C2	Quit within 2 years
P4	M	65–69	Highland	Urban	C2	Quit within 2 years
P5	F	50–54	Lothian	Urban	D	Quit within 2 years
P6	F	65–69	Tayside	Urban	D	Quit within 2 years
P7	F	45–49	Forth Valley	Urban	C2	Current smoker
P8	F	60–64	Highland	Urban	D	Current smoker

*Note*: Four males, four females, age 46–71 years, from six different health board regions, living in urban areas. Four current smokers and four of whom had quit within the last 2 years.

**Table 2:** Focus Group 2 participant characteristics

Focus group 2	Gender	Age range	NHS Health Board	Urban/rural area	SEG	Smoking status
P1	F	55–59	Greater Glasgow and Clyde	Rural	C2	Quit within 2 years
P2	F	45–49	Borders	Rural	D	Current smoker
P3	F	45–49	Grampian	Rural	D	Quit within 2 years
P4	F	55–59	Grampian	Rural	D	Current smoker
P5	M	60–64	Borders	Rural	D	Quit within 2 years
P6	M	60–64	Grampian	Rural	C2	Current smoker
P7	M	50–54	Borders	Rural	D	Quit within 2 years

*Note*: Four females, three males, age range 45–62 years, from three different health board regions, living in rural areas, three smokers and four of whom have quit within the last 2 years.

**Table 3:** Focus Group 3 participant characteristics

Focus group 3	Gender	Age range	NHS Health Board	Urban/rural area	SEG	Smoking status
P1	M	55–59	Greater Glasgow and Clyde	Urban	D	Quit within 2 years
P2	M	65–69	Greater Glasgow and Clyde	Urban	D	Quit within 2 years
P3	M	45–49	Greater Glasgow and Clyde	Urban	D	Quit within 2 years
P4	M	65–69	Forth Valley	Urban	C2	Quit within 2 years
P5	M	50–54	Grampian	Urban	D	Current smoker
P6	M	45–49	Grampian	Urban	C2	Quit within 2 years
P7	M	65–69	Forth Valley	Urban	D	Quit within 2 years
P8	F	60–64	Greater Glasgow and Clyde	Urban	E	Current smoker
P9	F	60–64	Forth Valley	Urban	C2	Current smoker
P10	F	60–64	Forth Valley	Urban	D	Current smoker

*Note*: Three females, seven males, age range 45–68 years, from three different health board regions, living in urban areas, four smokers and six of whom have quit within the last 2 years.

Our analysis identified three overarching themes in the data: (1) *Knowledge, awareness and acceptability of lung screening*, (2) *Barriers and facilitators to screening* and (3) *Promoting screening and implementation ideas*.

### 3.1 Knowledge, awareness and acceptability of lung screening

There is currently no national lung screening programme in the United Kingdom, although parts of NHS England are offering lung screening with LDCT.^25^ Participants were largely unaware of the concept of targeted lung screening:

No, I always thought that was something that happened if you develop, you know, or if they suspect you develop then you would have a check, otherwise nothing pre‐emptive…. (FG1, R5)

Most participants had heard of and participated in other forms of screening, including for breast, cervical and bowel cancer, and a few had been referred for lung checks for other reasons. They also described family members having had cancers picked up in this way, as well as their own experience of cancer:

For me, it really is, it's very important to be screened, especially breast, bowel, anything. I suffered myself from throat cancer ten years ago and I've been in remission for the past four years, so it's urgently important that people get this done, yeah. (FG3, R4)

When we described what lung screening would entail, participants were very supportive of this form of screening being available and welcomed the chance to have their lungs screened, with multiple participants saying it was ‘a great idea’. Participants were aware of the benefits of early (asymptomatic) detection and treatment:

I think the screening is a good idea to catch things earlier or to see if somebody's got the disease or whatever that they didn't know they had. (FG1, R7)

Participants also talked about the importance of screening early and not waiting until the ‘damage was done’, with discussions around age and smoking. It was suggested that raising awareness about lung cancer and lung screening should be introduced to school children, embedding knowledge of screening from an early age. Participants were also largely accepting of the fact that lung screening could pick up other issues,

R3: Anything that shows up as a side‐line to it is a benefit and I think most people would welcome it.

R1: Yeah, I 100 per cent agree with that.

R4: Yeah, totally agree with that one as well, an added bonus. (FG3, R1, 3 and 4)

However, there were certain caveats and conditions to participating in screening. The concept of targeted screening for smokers was problematic for participants, as they discussed other risk factors for lung cancer apart from smoking and that some people who never smoke go on to develop lung cancer:

There's other causes of lung cancer, it's not just smoking. (FG1, R7)

Participants introduced the potentially judgemental and stigmatizing nature of risk‐related eligibility to discussions. The importance of personal informed decision‐making and lack of coercion were also voiced by participants.

Facilitators asked participants about the role of smoking cessation advice in the lung screening process. Participants suggested that this would not put them off participating, although they reported a dislike of being pushed into stopping smoking or judged for their smoking, such that language and tone were important,

I think you have to have that balance … for people to [not] think, ‘oh, we're going there and we're going to have that shoved down our throat [i.e., “forced on us”]’. It has to be choice, but I think always giving people the appropriate choices if somebody is ready to stop, […] and giving them the information being there available, but I don't think making it part of something, because what would then happen is people would then think they're getting this shoved down our throat, we're getting judged for smoking, […] So I think, yes, it's good to have all the information, but it's how it's given. (FG1, R6)

Participants said that most people had received smoking cessation advice before, they already knew that smoking was harmful, and would not be offended by a health professional asking them about smoking. There was a strong sense among participants that the desire to quit smoking and action to quit was self‐motivated.

### 3.2 Barriers and facilitators to screening

It was clear that while there was strong support for the concept of lung screening with participants overwhelmingly in favour of it, a number of factors were raised which qualified their response—with the prospect of mediating the gap between reported intentions and performed screening behaviours in a real‐world scenario. Some of these same issues were potential motivators to participate in lung screening. Broadly, these can be represented under individual level and practical and system level factors.

#### 3.2.1 Individual level influences on screening intentions

On an individual level, there were a number of cognitive, psychological and emotional factors influencing screening intentions.

##### Psychological and emotional concerns

The most pronounced psychological and emotional concerns reported were fear and worry about cancer and interactions with health services. Fear of invasive procedures, disruption of their lives, waiting for results and the challenges of facing a cancer diagnosis were off‐putting for a number of people:

Maybe you don't want to know, maybe you don't want to have cancer so it's better, you know, just to kind of blunder on and not find out, so not even to go and to be scared of going. Also if you find out you have something wrong then you're going to have to change your lifestyle to make things better. (FG1, R5)

It's not knowing if you have the underlying issue or not, and then having to wait and then find out. I think that's maybe what puts a lot of people off not actually doing it. I think they're just maybe prepared, until they get a scare themselves and then go through a test, they're willing to just bypass it. (FG2, R5)

A lot of people are scared to come forward in case that the results of a test are positive. A lot of people don't want to know. And while they feel okay and there are no symptoms, that's fine. And then for someone to say to them, oh, by the way, you have this or that, it's quite scary for some people. (FG3, R3)

While participants (such as respondent 3 above) spoke of fear of the unknown, they were motivated to see what damage had been done to their lungs, showing the complexity of these thoughts in influencing behaviour,

I wonder what stage my lungs are really at? And I'm sure other people think like that as well. […] It's like they know but we don't know and I think we should know. (FG3, R3)

Other issues raised in the focus groups related to problems engaging the older generation and males in particular, who they suggested were often stoical in their approach to health and illness and reluctant to burden health services—or they may see no point in screening when they are able to continue functioning. For some participants, the intention to be screened was related to being conscientious citizens and prioritizing one's health above other competing demands:

You know, most people are concerned for their own health, they want to be healthy, they don't want to be a burden to the doctor or the NHS. But if you've got something wrong, early intervention is the answer. (FG3, R2)

##### Perceived risk and fatalism

Perceived risk of lung cancer also appeared to influence people's screening intentions. Many people felt that their relatively young age and lack of symptoms meant that they were unlikely to have lung cancer and so screening was not relevant to them, though this was tied up with fear and a sense of fatalism in a complex way:

I'm fit and I'm healthy. If I go and get it and it comes back it's chronic or it's terminal, fine, it's only terminal for as long as I'm going to last. (FG3, R4)

I feel myself slightly younger than everyone here that I see screening as a slightly older person's thing or a female thing, and you don't have to worry about it until you're like a certain age or something like that. (FG1, R5)

Perceived risk was also associated with family history and advancing age. Bad experiences of cancer in the family were discussed as both a source of avoidance of screening and a motivator to take part to avoid late detection and a poor prognosis:

Yeah, I've been for a cervical one because unfortunately on my mum's side of the family it seems to run in the family. So, my mum and my older sister have all had hysterectomies at a young age … You have to go for these screenings, especially if you find it runs in your family. Even if it doesn't run in your family, the older we get, we are getting older and it's all inside as well so it's good to know what's going on. (FG2, R5)

As above, fatalism was also evident in discussions among those who had smoked heavily and felt that lung cancer was unavoidable:

At the end of the day, I've smoked since I was 15, so what damage is done [, is done]. (FG3, R4)

##### Stigma and judgement as a barrier

A common theme across all focus groups was the role of judgement of smoking behaviour and perceived stigma as a barrier to presenting for lung screening:

The stigma of people who smoke is very real and they're made to feel uncomfortable. Although for years and years we were encouraged to do it, it was modern, it was yuppy, it was everything you wanted. It was cool, sophisticated, and yet now smokers feel … Well, I don't know, I'm only speaking for myself, I feel as if, oh well, it's your own fault, you caused it. I did cause it, but I was encouraged to cause it. (FG3, R4)

Participants were also conscious of the cost of screening and did not want to be seen to take advantage of the system:

No, but you could get members of the public being quite judgemental because I smoke so I know the health concerns, so if I'm choosing to do a risk‐taking behaviour, shall we say, I'm presuming this is something that the taxpayer is going to pay, so I'm thinking somebody might think why should I get tested for something that I'm putting myself at risk for. That might put people off. (FG1, R6)

##### Mistrust of healthcare professionals and services

A number of participants reported good experiences with health services and a proactive approach to their health, driven by an awareness of the benefits of early detection. However, poor experiences with health services and healthcare professionals compromized trust and limited faith in services for some participants, and therefore an avoidance of any kind of interaction with them:

I wouldn't trust my GP in that case to recommend, you know? Maybe he's biased, he's thinking, okay, that guy's smoking so he's just wasting time and money anyway so I won't recommend him. (FG1, R5)

Participants in two of the groups discussed the fact that GPs are often overworked and a perception that patients' smoking results in GPs not investigating issues adequately or treating them fairly. By‐passing GPs and attending screening through an independent screening programme was seen as a positive thing. On the other hand, some participants reported good experiences and welcomed an endorsement from their GP, with one person commenting, ‘It's formal coming from your doctor’ (FG1, R5).

#### 3.2.2 Practical and system barriers

A number of practical barriers to attending for screening were raised by participants. These included time off from work leading to loss of money, distance of travel to an appointment and access issues:

If you got a letter in saying, oh, you've got to go at ten o'clock in the morning and it's hardly worth going to work before that because you have to travel or whatever, so you might end up losing four hours' pay, you know what I mean? So if there's an incentive to encourage people for them not to lose money, I think you would get over 90 per cent of people would do it. (FG1, R3)

Living in remote areas of Scotland, while having local access to primary care, was also voiced as an issue in terms of accessing secondary care, which could be a problem for a hospital‐based lung screening:

We're lucky in [place] that we've got a hospital and it covers the whole of the north but there are lots of places that … and there are a lot of old people that can't get to it. (FG1, R2)

Participants also had some concerns about the ability of health services to meet the demands of screening in terms of providing an accessible and timely service. Delays and waiting for test results were a source of worry for people and there was a common reported perception of the NHS as an under‐resourced and over‐stretched service.

Having outlined what the screening process would entail, participants did not voluntarily raise any concerns about radiation exposure, over‐diagnosis or invasiveness of the LDCT procedure. Participants were supportive of a ‘proper’ check of the lungs, including identification of nodules to be monitored or other incidental findings, particularly when they had existing lung problems.

### 3.3 Promoting screening and implementation ideas

There was considerable discussion in each focus group on the acceptability and accessibility of screening, leading to suggestions of ways of promoting and implementing screening to increase participation. Participants' views on accessibility were of particular interest due to their socioeconomic status and geographical diversity. Suggestions related to the cognitive and psychological barriers to screening as well as practical issues.

#### 3.3.1 Managing fears and expectations

To address fears around lung cancer, participants suggest that there is an emphasis in any information materials or advertising campaigns on positive messaging. Examples were given of being able to live to play with grandchildren and harnessing the successes of other screening programmes:

I think to get people to go to screening, you need to publicise the success of other screening programmes, whether it's bowel, breast, cervical, whatever it is. Publicise how successful these are and really go for it and say, right, this is the next step in the screening and it's going to be a lung screening. (FG3, R1)

Participants said that an invitation to screening should be encouraging without being coercive, should not mention the word ‘cancer’ too much and should not imply judgement about smoking behaviour:

There's always going to be a worry anyway. But seeing the word ‘health check’ you would get more people to go. The word cancer and people just say, well I don't want to know. (FG2, R3)

#### 3.3.2 Improving accessibility

In addition to the implementation ideas given above, there was a very clear message from the focus groups when it came to addressing any access barriers to screening, relevant to both deprived and remote and rural communities—the use of mobile units. This, it was typically felt, would remove screening from a clinical environment, and bring it closer to local communities.

So, if there was screening available as a mobile unit or some sort of drop in people probably would be more willing to go and fit it in. It's just the same as these COVID jabs we've all had to do; if they were more available, a lot more people would do it. (FG2, R3)

This is where the mobile vans come in, you know? They can drive to these remote areas, especially in Scotland, when they go further north. We've got two people from [place], […] some of the places are very remote so…. (FG1, R1)

While those in the rural‐dwelling focus group were unanimously in favour of mobile units, support for them was not confined to the rural group. Mobile units were supported as a way of engaging with marginalized groups, such as those who sought to avoid hospitals.

Supporting people who would lose pay if they attend a screening appointment was suggested as a statutory right, with employer support:

I think your employer should get help if people need time off to go to these things, they should be encouraging and get paid for going to them, you know, get paid for so many hours, if you need so many hours off to go and get these tests done, you shouldn't lose your pay, because if you lose your pay, a lot of people will not go. (FG1, R3)

Another suggestion for increasing participation was to ‘flood’ the public with information about screening through all media outlets about screening and engage with local communities, particularly the harder to reach groups, through ‘men's sheds’, workplaces, community hubs for older people as well as speaking to school pupils to normalize screening:

And taking the fear out of it by having all different age groups talking about it … It's trying to get the message out there, people need to start screening themselves from an age where they're invited to do so, and not be fearful of it. It's trying to get around that age group and succeeding with it, and hoping that future generations do go for their screenings when they're invited to do so. (FG2, R6)

## 4 DISCUSSION

### 4.1 Summary

This focus group study is the first of its kind to ascertain the views of Scottish residents on a potential LDCT lung screening programme and identify likely barriers and facilitators in this context. Knowledge of lung screening was low among the focus group participants, although they showed awareness and personal experience of other screening programmes. Participants were very supportive of the idea of lung screening and harnessed the early detection narrative to discuss the importance of taking part. Participants reported the process of lung screening via a LDCT scan was acceptable. Two key barrier types were identified: individual level influences on screening intentions, and practical and system barriers. Within the individual level factors, emotional and psychological concerns related to fear of a cancer diagnosis, mistrust, fatalism and perceived stigma were dominant in focus group discussion. For some, screening was part of being a health conscious citizen and prioritizing health matters, while others based their decision‐making on their perceived risk. A number of potential practical barriers to lung screening participation were mooted that were particularly relevant for people living in deprived and rural communities. These included travel, cost, time and competing priorities. Maximizing accessibility was also key in the discussion and a distinct recommendation for future programmes.

### 4.2 Comparison with existing literature and theory

There is a strong consistency in our findings with the growing body of evidence looking at attitudes to screening and screening behaviour, across a range of screening programmes and for lung screening in particular, in the United Kingdom and beyond.^26^, ^27^, ^28^ LDCT screening for lung cancer is largely unheard of in Scotland but has a high degree of acceptability more broadly, or among those who have participated in screening.^27^, ^29^, ^30^ There was an evident awareness among participants of the benefits of early detection and thus support for lung screening, in line with other research.^17^, ^31^

Fear was identified as one of the most common psychological barriers to lung screening, which is reflected in the literature in both survey and qualitative explorations of attitudes to screening.^28^, ^32^, ^33^ Linked to this was a sense of fatalism or predicted fatalism among older generations, also mirrored in comparable studies of lung screening.^29^, ^34^ While there is evidence to suggest that the fear and anxiety associated with lung screening participation is transient^35^ and can be a motivating factor to be screened, quit smoking and even other cancer‐preventing health behaviour change,^36^, ^37^, ^38^ it is still vital to minimize this emotional response by addressing and managing it. Positive messaging in the promotion of screening, such as sharing the treatment successes and mortality gains from early detection, is one potential step in approaching this.

Notions of risk were a key component of our group's considerations of whether or not someone would take part in screening, often related to not experiencing symptoms and having stopped smoking. Perceived risk has been identified widely in the literature to explain decision‐making in relation to screening and help‐seeking for symptomatic illness.^28^, ^33^, ^39^, ^40^ Risk and decision‐making are discussed further below.

Perceived judgement and stigma related to smoking featured in focus group discussions. This is widely evident in the literature, along with self‐blame.^31^, ^38^, ^41^ Stigma has been identified in the literature as a barrier to help‐seeking for signs of lung cancer and it seems this also applies to screening participation.^42^ Related to this was a sense of fatalism—participants in our study did not consider they would blame themselves if they developed lung cancer, but some did feel that the damage was already done and screening could not change that.^17^, ^29^, ^38^ However, this was not a clear barrier. For some, it was a good reason to detect any inevitable lung cancer at an early stage, suggesting that issues such as fear, blame and fatalism are complex and operate on a pendulum when prompting action or inaction.

Discussions of stigma inevitably moved onto smoking cessation. Smoking cessation advice was broadly acceptable to our participants, but only if delivered in a noncoercive way; again, other studies have had similar findings.^17^, ^43^, ^44^, ^45^ In our focus group study, it appeared that participants may have become desensitized to smoking cessation advice. This has implications when considering brief interventions for smoking, and would need further exploration to understand whether small prompts may be enough to stimulate action among people who have intentions to quit smoking. There is evidence to suggest that brief interventions can be effective.^46^, ^47^ There is also emerging evidence that incorporating smoking cessation advice into lung screening is effective—seeing images of lungs has been a strong motivator to quit smoking^36^ as well as being central to the long‐term cost‐effectiveness of screening.^13^

Some participants in our focus groups described a difficult relationship and a level of mistrust in interacting with health professionals and services, often related to poor past experiences or a sociocultural divide. Such perceptions are often ingrained in more deprived communities and are often reported in the literature—relating not only to lung screening,^17^, ^32^ but beyond to studies of help‐seeking behaviour and doctor–patient relationships.^48^ Again, this is a complex feature of health service engagement and can be linked to other barriers such as low self‐efficacy, low health literacy and power dynamics, which can be particularly divisive in terms of equity in access to health care for people in disadvantaged groups.^48^, ^49^, ^50^ Development of interventions to repair broken relationships with the health service, a perceived authoritative institution, and other methods to improve accessibility such as communication training for professionals, targeted awareness campaigns and community engagement strategies can address inequities in access to screening services.^51^ Primary care has an important role to play; current workforce shortages need to be addressed, and solutions identified which do not generate significant extra burden for primary care staff—one example is streamlining procedures to identify high‐risk patients from practice data.^7^, ^8^ It also seems logical to harness the successes of implementation strategies for other cancer screening (e.g., the UK's bowel screening programmes).^52^, ^53^

In addition to the psychosocial issues discussed by focus group participants, practical barriers to participation in lung screening including time, cost, travel and competing work or other commitments, were also mooted. Practical barriers are commonly reported throughout the evidence base related to engaging with screening, with a suggestion that these barriers are heightened in more deprived groups.^27^, ^32^, ^46^

Von Wagner et al.^50^ have developed a model of screening behaviour, accounting for a range of factors identified in relation to wider health behaviours, such as health identity and self‐efficacy, that can be usefully mapped onto the findings of this study. Similarly, Robb^54^ has developed the I‐SAM model to understand screening participation. Application of these models to follow‐up interviews as part of the planned pilot lung screening study in Scotland will be enlightening to confirm the salience of these to screening participation and nonparticipation.

There is an abundance of early cancer detection research exploring how people appraise bodily changes, evaluate risk and decide to seek medical help, as well as conceptual models to understand these processes.^55^, ^56^, ^57^ There is also some utility in applying these to screening behaviour, often in the absence of symptoms, to understand nonparticipation in screening. For example, Kummer et al.'s^37^, ^39^ cognitive heuristics for help‐seeking for cancer symptoms may act as prompts or inhibitors to participate in screening. Understanding these factors in the context of deprived populations adds further considerations that may compound behaviour in terms of available resources (cognitive, psychological and practical), competing demands and permeability of services.^48^, ^49^

In terms of ideas to overcome barriers, participants focused primarily on positive messaging in information materials and advertising, and accessibility through the provision of mobile screening vans similar to those used in breast screening or for Covid‐19 vaccination. Positive and nonjudgemental messaging focusing on the gains on offer from screening and early detection has also been found elsewhere and incorporated into the pilot lung screening provision.^58^, ^59^ Travel to access screening services as well as fear of hospital environments have also been identified in the literature, and evidence evaluating the use of mobile screening vans to address these issues is beginning to accumulate.^12^, ^60^

### 4.3 Strengths and limitations

This set of focus groups provides a rich data set with an in‐depth discussion of the concept of lung cancer screening and anticipated barriers and facilitators. Focus group participants were a self‐selecting group who are accustomed to taking part in research studies, and who received a financial incentive. However, this form of recruitment strategy does allow access to people from a range of different backgrounds, including both deprived and rural areas of Scotland that may otherwise have been challenging to recruit. Three of the 24 participants were from ethnic minority groups, reflecting the general demographic in the Scottish population. Focus groups were conducted online. Video conferencing software facilitates easy interaction with people from around the country but can pose challenges. These include failing technology, building rapport and ensuring balanced participation within the group simply by being in a room together and acknowledging social cues. It was also necessary to consider that participants were talking about a hypothetical scenario and their intended behaviour, something we know does not simply translate into action.^61^ Speaking to people who have taken part in screening or chosen not to take part is also essential to further understand the relationship between intention and behaviour.

We also reflected on the group dynamic together with the nature of our role in conducting the focus groups, and whether this was likely to influence people in agreeing with and supporting the concept of lung screening. However, the open nature of questioning, reminding participants that we genuinely wanted to hear their views, and the self‐selecting group of individuals who were quite assured in their own responses, suggested that we did not shape this narrative.

### 4.4 Implications and future work

This study informs the development of strategies to improve uptake and informed choice in lung screening. It is essential to understand people's health beliefs and behaviours and to target the barriers to implement a patient‐centred service using a theory‐driven approach.^62^ This work adds to a growing evidence base shedding light on the behavioural aspects of screening participation and will inform the design and implementation of a new lung screening pilot in Scotland^40^, ^45^, ^46^ (see Box 2 for implementation ideas generated from this work). Minimizing practical barriers is also likely to be instrumental in improving participation and addressing inequity in access to screening. As such, information materials, methods of communication and the design of the process involved in screening (e.g., minimizing the steps, time commitment and waiting intervals), and sensitive, supportive messaging that addresses stigma and fear are all important components of a pilot to break down some of the known barriers. Drawing on the similarities with research based on other UK pilot studies, we are modelling optimized study and participant materials.^59^, ^63^, ^64^ While there is consistency in the findings compared with existing work, it was important to explore the Scottish context with a diverse sample of participants to consider how rurality and deprivation presented any unique issues. Understanding ethnic variations in views on lung screening participation will also be important to ensure equitable provision and uptake of screening. A high burden of multimorbidity is also characteristic of the Scottish population and should be examined in future studies.

Box 2: **Potential strategies for implementation of lung screening**

- Minimize steps in the screening process to lower opportunities for delays and associated distress
- Avoiding unnecessary travel to scanning facilities with the chance to discuss screening concerns, address fears and perceived stigma, and facilitate informed decision‐making
- Use of mobile screening vans as a ‘one stop shop’ to address resource constraints and travel issues for people living in deprived and rural areas
- Consider covering the cost of travel expenses to screening facilities to ensure equitable access
- Incorporating discussion of fears associated with screening into information materials
- Ensuring positive messaging with nonjudgemental language around smoking behaviour
- Ensuring a timely and sensitive approach to smoking cessation advice

## 5 CONCLUSIONS

This focus group study has already identified several perceived individual, practical and system barriers and facilitators to participation in a pilot lung screening programme in Scotland using LDCT. While our results resonate with existing literature in this field, they will be helpful in addressing factors which are especially important in Scotland if it is to embrace lung cancer screening—reducing health inequalities, engaging deprived populations and ensuring access in remote and rural areas. The findings will inform the design and implementation of a Scottish pilot lung screening study.

## AUTHOR CONTRIBUTIONS

All authors apart from Jasmin Rostron and Lynsey R. Brown contributed toward the design of the focus groups and helped develop study documents. Debbie Cavers and Mia Nelson conducted the focus groups. Debbie Cavers, Mia Nelson and Jasmin Rostron coded and analysed the transcripts. Lynsey R. Brown read and commented on the analysis. Debbie Cavers drafted the manuscript and all authors edited and refined subsequent drafts.

## CONFLICT OF INTEREST

F. S. declares that the Universities of Dundee and St. Andrew's received funding from Oncimmune for the Early Detection of Cancer of the Lung Scotland (ECLS) trial from 2013 to 2021. The remaining authors declare no conflict of interest.

## ETHICS STATEMENT

The LUNGSCOT focus group study was approved by the University of Edinburgh Medical School Ethics Committee on 19 March 2021, reference 21‐EMREC‐002. All participants gave consent before taking part in the focus groups.

## Supporting information

Supporting information.

## Data Availability

Data are available on request to the authors.
