# Supplementary material for: Optimizing the implementation of lung cancer screening in Scotland: Focus group participant perspectives in the LUNGSCOT study
Source: Health Expect. 2022 Oct 20;25(6):3246–58. doi: 10.1111/hex.13632 (PMC9700133; doi:10.1111/hex.13632)
Supplement: Supplementary file 1 — Supporting information. [file HEX-25--s001.docx]

Lung health check focus group study – topic guide

[Check for questions and confirm verbal consent using the consent form and asking people to answer yes or no.]

Introduction: Welcome to everyone and thank you again for agreeing to take part in this focus group to discuss your views on the best ways to introduce a lung health check and lung screening via local general practices. For someone undergoing screening, this would involve having a lung health check by answering a questionnaire about their general health, smoking history and family history. For those where there is concern for the health of their lungs, they would be referred to the hospital for a CT scan using low dose radiation to look for early signs of lung cancer or other lung conditions. Picking up lung cancer at an early stage can mean that people have a much better chance of being treated successfully and surviving lung cancer.

For today’s discussion, it is important that everyone has a chance to have their say and that we are all respectful of other people’s opinions. You don’t have to share anything that you are not comfortable with and you are all free to stop at any time if you wish. There are no right or wrong answers here but we can learn a lot from listening to your views and experiences with health care to make sure we design a service that will meet everyone’s needs. Does anyone have any other questions before we begin? Is it okay if I switch on the recorder to begin the discussion?

Focus group topics

**Views and understanding of screening programmes**

Firstly, we are interested to hear your views about screening programmes in general such as bowel screening or breast screening where people have checks to look for early signs of cancer.

- What do you understand about health screening programmes?

- What do you know about why screening takes place?

- Do you think people should participate in screening?

- Has anyone heard anything about lung screening programmes being introduced?

- What do you think about this?

**Barriers and facilitators to screening participation**

- Can you think of any reasons why people would choose to take part in screening?

- Can you think of any reasons why people may not take part in screening?

- Do you think people choose not to take part? Why?

- What other reasons might there be that people do not take part in screening?

**Barriers and facilitators to lung screening participation**

- Do you think there might be any particular reasons why people might not take part in a lung health check/ screening programme?

- What might make it difficult for people to take part?

- What might make it easier for people to take part?

- In your local community, can you think of any particular issues that might stop people from wanting to take part in lung screening?

**Feasibility and acceptability of lung screening via general practice**

- Do you think asking people to come into their general practice for a lung health check is a good idea? Why?

- Do you think the location makes a difference?

- Where else do you think lung health checks and CT screening should take place?

- would you prefer to go somewhere else rather than the GP?

- Who do you think would be the best person to carry out the lung health check at the GP practice? The GP or the practice nurse or someone else?

- Do you think it would be a good idea to ask people to come in especially for the lung health check or to do it while they are there for something else?

- How do you find going for health-related appointments? E.g. GP, hospital or dentist?

- How do you feel about going? Comfortable/uncomfortable? Relaxed/nervous?

- Is there anything you like or don’t like about it?

- How would you feel if you received a letter at home asking you to come in for a lung health check?

- Is there any particular wording that you think would be most appropriate to use in the letter?

- What do you think of terms like ‘lung health check’ or ‘lung MOT’ rather than lung screening or lung cancer screening?

- How would you feel about being identified as a smoker and asked about smoking behaviour by your GP?

- How would you feel about being given advice about stopping smoking?

- If you imagine receiving one of these invitations for a lung health check yourself, what factors would influence whether or not you went for the check?

- Do you think the amount of information sent to people could influence whether or not they take part?

- What would be considered ‘too much’ information or otherwise off-putting?

- How would you feel if you were asked to go for a CT scan to look for a possible lung cancer or other problems with your lungs?

- Is there anything that could make this easier for you?

- What do you think about the fact that it is a two-step process, a lung health check and then referral to the hospital for the scan?

- Do you think going for the scan would have an emotional impact on you?

- What would having a clear scan mean to you?

- What would you want to happen after you got a clear result?

- how you feel about the fact that you might not be followed-up?

- how would you feel about another issue with your lungs such as emphysema or lung nodules being picked up and needing follow-up?

- has anyone heard of ‘lung nodules’ before? [Sometimes the scans pick up small nodules that may or may not need follow-up.]

- Can you think of any other ways of delivering the screening programme that might make people more likely to take part?

**Exploring issues of rurality and deprivation**

- Do you think where people live makes a difference to whether or not they would take part in lung screening? i.e. living in the city versus more rural areas?

- Can you think of anything that might put off people that you know or who live in your local area?

- Can you think of any ways of trying to encourage people you know who might be reluctant to come in for lung screening?

- What do you think the particular barriers might be?

**Designing the optimum service**

- Does anyone have any ideas for a lung health check service to make sure that it reaches people across the country and targets those who are less likely to participate?

- What do you think this kind of screening programme would look like?

**Closing**

- Does anyone have any other comments or suggestions that haven’t already been covered?

Thank you very much for your time. Can I also remind you that if anyone has any concerns about their lungs, that they make an appointment to see their GP as soon as possible.
